# 817. Caspofungin versus Anidulafungin in Patients with Candidemia: Retrospective Comparative Study

**DOI:** 10.1093/ofid/ofad500.862

**Published:** 2023-11-27

**Authors:** Reem Elajez, Dana Bakdach, Sara Al Balushi, Tasneem Abdallah, Ahmed Zaqout, Rand Alattar, Waleed Awouda, Godwin Wilson, Hussam Abdelrahman Alsoub

**Affiliations:** Hamad Medical Corporation, Doha, Ad Dawhah, Qatar; Hamad Medical Corporation, Doha, Ad Dawhah, Qatar; Hamad Medical corporation, Doha, Ad Dawhah, Qatar; Hamad medical corporation, Doha, Ad Dawhah, Qatar; Hamad Medical Corporation, Doha, Ad Dawhah, Qatar; Hamad Medical Corporation, Doha, Ad Dawhah, Qatar; Hamad Medical Corporation, Doha, Ad Dawhah, Qatar; Hamad Medical Corporation, Doha, Ad Dawhah, Qatar; Hamad Medical Corporation, Doha, Ad Dawhah, Qatar

## Abstract

**Background:**

Echinocandins are recommended as initial treatment for candidemia. The safety and efficacy of both anidulafungin and caspofungin are well established, yet; no direct comparison was made pertaining to their efficacy and safety. This was a retrospective study to compare efficacy and safety of anidulafungin versus caspofungin in patients with candidemia.

**Methods:**

All adult patients with candidemia who were treated with either anidulafungin or caspofungin for ≥ 5 days, over a period of 6 years were retrospectively included. Baseline demographics, infection characteristics and patient courses were assessed. The primary end point was global response defined as clinical and microbiological success at the end of treatment duration.

**Results:**

A total of 171 patients received either anidulafungin (n = 135) or caspofungin (n = 36) based on physician preference. The most common isolated candida species were *candida glabrata* (31.6%) followed by *candida albicans* (25.1%). Around 6% had chronic liver disease, and source control (mainly catheter removal) was achieved in 91.8%. Baseline characteristics were comparable between the two groups suggesting similar risks and treatment response. Response rates were similar among both groups with the primary outcome of global response being not significantly different (50% for caspofungin group vs 65.2% for anidulafungin, p=0.666). Similarly, no differences between the two groups were observed in terms of 90-day all-cause mortality (p=0.405) and hepatic safety profile defined as elevated liver function tests ≥ 3 times from baseline (p=0.521)

**Conclusion:**

Our data suggested that among patients with candidemia, there was no difference between anidulafungin and caspofungin for the primary endpoint of global response. Both studied echinocandins had similar rate of 90-day all-cause mortality and liver safety profile.

**Disclosures:**

**All Authors**: No reported disclosures

